# Emergence of a medley of invasive fungal infections amidst the coronavirus disease 2019 (COVID-19) pandemic in India

**DOI:** 10.1017/ash.2021.198

**Published:** 2021-11-02

**Authors:** Sudhan Rackimuthu, Hiba Khan, Anmol Mohan, Reem Hunain, Behram Khan Ghazi, Mohammad Mehedi Hasan, Ana Carla dos Santos Costa, Shoaib Ahmad, Mohammad Yasir Essar

**Affiliations:** 1 Father Muller Medical College, Mangalore, Karnataka, India; 2 Dubai Medical College, Dubai, United Arab Emirates; 3 Department of Medicine, Karachi Medical and Dental College, Karachi, Pakistan; 4 Kasturba Medical College, Manipal, India; 5 Punjab Medical College, Faisalabad, Pakistan; 6 Department of Biochemistry and Molecular Biology, Faculty of Life Science, Mawlana Bhashani Science and Technology University, Tangail, Bangladesh; 7 Division of Infectious Diseases, The Red-Green Research Centre, BICCB, Dhaka, Bangladesh; 8 Faculty of Medicine, Federal University of Bahia, Salvador, Bahia, Brazil; 9 Department of Paediatrics, DHQ teaching hospital, Faisalabad, Pakistan; 10 Kabul University of Medical Sciences, Kabul, Afghanistan

*To the Editor*—The coronavirus disease (COVID-19) outbreak caused by the severe acute respiratory syndrome coronavirus 2 (SARS-CoV-2) has affected the whole world while disrupting global health. Even with the second wave of the pandemic now abating slowly but steadily, cases of fungal infections among COVID-19 patients and those who have recovered are imposing an extra burden. While the country was already reporting a staggering number of ‘black fungus’ (mucormycosis) among COVID-19 victims, reports of the emergence of ‘white fungus’ and subsequently a case of ‘yellow fungus’ and most recently ‘green fungus’ have sparked further tension.^1^

Mucormycosis, caused by *Mucormycetes*, a type of mold present in damp environments like soil or compost, is a rare and lethal fungal infection commonly affecting immunocompromised individuals. It is characterized by tissue necrosis and targets the sinuses, lungs, brain, and skin. A study on mucormycosis cases found an overall mortality rate to be 54%, which may be even higher when including underlying comorbidities and coinfection with COVID-19.^2^

*Candida auris*, an *Ascomycetes* yeast, also called ‘white fungus’ is an emerging global threat that has multiple cases reported in India amid the COVID-19 pandemic. This nosocomial infection particularly infects patients with low immunity in the intensive care unit. *C. auris* transmission in hospitals during the COVID-19 pandemic in India poses a high risk due to their limited facilities for fungal identification and antifungal susceptibility testing.^3^ Owing to its multidrug resistance and rapid transmissibility in hospital settings, it is also called a “superbug fungus” that causes bloodstream infections with a high mortality rate.

*Aspergillus flavus*, belonging to the Aspergillus family, is suspected to be the ‘yellow fungus,’ due to its yellow-colored mold. This thermotolerant fungus generally affects the lungs of immunocompromised individuals. Symptoms of coinfection with aspergillosis and COVID-19 include fever, chest pain, cough, hemoptysis, and breathlessness.^4^ The ‘green fungus’ is also caused by a member of the Aspergillus family. Most aspergilli detected are azole resistant, which can lead to challenges in the management and impending broader antifungal resistance.

Steroids are commonly used in treating patients with moderate or severe COVID-19 by countering the systemic inflammatory response.^5^ However, the use of steroids decreases the overall immune response of the patient making them more vulnerable to secondary infections like that of a fungal etiology. The increased use of zinc supplementation in COVID-19 patients has also been highlighted as a possible contributor to the surge in invasive fungal infections.^6^ To make matters worse, as a result of the deteriorating healthcare infrastructure and resources caused by the ongoing COVID-19 pandemic, many patients are self-medicating without proper knowledge with over-the-counter, easily accessible drugs and many patients are using oxygen therapy without proper hygiene. These factors have become reoccurring concerns, particularly in India, because they are among the most common preventable causes of COVID-19 patients developing super-added fungal infections. In addition, fungi tend to manifest more commonly in individuals with uncontrolled diabetes.^7^ India has ∼77 million diabetic patients, and augmented by widespread noncompliance to medication, this vulnerability poses another serious concern.^8^ Contaminated water used in humidifiers for oxygen therapy, industrial oxygen, unsterilized medical equipment, prolonged use of the same masks, and tubing are also strongly believed to cause fungal infections.^9^ The unhygienic environment and poor living standards in the slums and rural areas of India likely play a role in this fungal outbreak. The climate of South Asia, with high temperature and humidity, is also thought to contribute to the favorable growth of these fungi.^10^

The demand for antifungal medications has risen because of fungal infections in COVID-19 patients. A severe shortage of the amphotericin B, which is the first-line treatment of choice for mucormycosis, has developed, increasing mortality and further panic. The fear of being unable to attain the required medications has caused people to hoard drugs, further contributing to the shortage. This dearth of antifungal medication has created a black market for drugs that were already too expensive for most people to afford.

With the emergence of candidiasis and aspergillosis cases, the paucity of antifungal drugs has been further aggravated. India’s continuous battle with COVID-19 has resulted in hospitals running out of beds, ventilators, and oxygen cylinders, which has continued to strain the healthcare budget and infrastructure. Additionally, treating most invasive fungal infections is challenging because it requires multidisciplinary expertise. In an overwhelmed healthcare system, finding surgical facilities with postoperative care for patients suffering from fungal and COVID-19 coinfection can pose another logistical nightmare. However, the crisis could be averted with the help of the recommendations listed in Table 1.

**Table 1.** Recommendations for Prevention and Effective Management of Invasive Fungal Infections During the COVID-19 Pandemic

1	Using masks and wearing shoes, long trousers, long-sleeved shirts, and gloves while visiting construction sites or handling soil, moss, or manure
2	Maintaining personal hygiene by thorough scrubbing while taking a bath and maintaining a clean environment with preferably cool temperature with reduced humidity
3	Being aware of signs and symptoms of invasive fungal infections, especially in individuals who are diabetic, immunosuppressed or previously afflicted with COVID-19
4	Using of steroids with minimal required dose and duration as recommended by a physician
5	Maintaining sterile and clean water and piping for humidifiers and vents
6	Use of antifungals, zinc, and other drugs appropriately as part of the COVID-19 armamentarium, considering disease severity and implicated causative organism, as recommended by a physician
7	Mobilizing and increasing production of antifungal drugs to prevent shortages

With cases of fungal and COVID-19 coinfections still being recorded all over India, it is imperative to exercise caution and to continue to adhere to preventive guidelines. Physicians should be cognizant of the likelihood of invasive secondary fungal infections in patients with COVID-19 infection, especially in those who have pre-existing risk factors. Physicians should be able to detect and treat these infections early to help reduce mortality and morbidity. It is also beneficial to address the fungal infections by name and by the implicated causative organism rather than color to avoid confusion and altercations among the general public and physicians to help with an accurate diagnosis, treatment, and prognosis.
